# A Randomized Controlled ‘REAL‐FITNESS’ Trial to Evaluate Physical Activity in Patients With Newly Diagnosed Multiple Myeloma

**DOI:** 10.1002/jcsm.13793

**Published:** 2025-04-08

**Authors:** Esther Dreyling, Jan Räder, Mandy‐Deborah Möller, Gabriele Ihorst, Sina Wenger, Antonia Pahl, Jann Arends, Georg Herget, Peter Deibert, Ralph Wäsch, Monika Engelhardt

**Affiliations:** ^1^ Department of Hematology, Oncology and Stem Cell Transplantation, Medical Center University of Freiburg, Faculty of Medicine University of Freiburg Freiburg Germany; ^2^ Clinical Trials Unit, Faculty of Medicine University of Freiburg Freiburg Germany; ^3^ Department of Orthopedics and Trauma Surgery, Faculty of Medicine University of Freiburg Freiburg Germany; ^4^ Institute for Movement and Occupational Medicine, Faculty of Medicine University of Freiburg Freiburg Germany

**Keywords:** multiple myeloma (MM), physical activity (PA), quality of life (QoL), randomized clinical trial (RCT), World Health Organization (WHO)

## Abstract

**Background:**

Multiple myeloma (MM) is the second most common haematological malignancy. The predominantly older patients often suffer from comorbidities that impair their quality of life (QoL). Physical activity (PA) can be beneficial for cancer patients, but less evidence exists in MM. This randomized controlled trial (RCT) compared an exercise group with World Health Organization (WHO)–compliant PA (150 min aerobic exercise and 2 resistance training‐sessions/week) vs. activity as usual (control group).

**Methods:**

Thirty‐four newly diagnosed consecutive MM patients were randomized 1:1 to exercise vs. control groups. Guided training (2×/week) was performed for 3 months during bortezomib–cyclophosphamide–dexamethasone (VCd) induction. PA was monitored using smartwatches and diaries. Demographics, osteolytic lesions, infections, fatigue, depression, and biomarkers (albumin, creatine kinase, C‐reactive protein, high‐density lipoprotein, low‐density lipoprotein and pro‐brain natriuretic peptide) were compared in exercise vs. control cohorts. VCd‐tolerance, response, ‘timed‐up‐and‐go‐test’ (TUGT), Revised Myeloma Comorbidity Index (R‐MCI), QoL (SF‐12 questionnaire), event‐free survival and trainer assignment during the training period were assessed (13 tests at baseline, during VCd and end of treatment [EOT]).

**Results:**

The exercise group was more than twice as active as the control group, with an average aerobic activity of 162 versus 68 min/week, respectively. Trainer‐guided muscle‐strengthening exercises were performed 2×/week in the exercise group, in line with WHO recommendations. These data were monitored via smartwatches and training diaries. PA proved to be safe: No exercise‐related SAEs or accidents occurred. The study adherence was 94% (32/34). In the exercise versus control group, AEs to VCd induction (6% vs. 25%), therapy intolerance (6% vs. 25%) and hospitalization (31% vs. 50%, respectively) occurred less frequently. VCd‐dose adjustments in the exercise vs. control group were significantly less needed (6.3% vs. 37.5%, respectively). At EOT, patients in the exercise group showed less fatigue (6% vs. 75%), less depression (6% vs. 44%), better TUGT (6 vs. 11 s, respectively), improved R‐MCI and QoL compared to the control group. Grip strength (right hand: 73–82 lb; left hand: 68–72 lb) significantly improved from baseline to EOT in the exercise group. Biomarkers did not significantly differ in both groups, but response to VCd‐induction and event‐free survival were improved in the exercise group, however, without reaching statistical significance.

**Conclusions:**

PA in MM patients during induction is feasible and can improve fatigue, depression, TUGT, grip strength, comorbidities and QoL. More sport intervention offers are warranted to advance exercising in MM.

**Trial Registration:** drks.de: DRKS00022250

## 1 Introduction

Multiple myeloma (MM) is the second most common haematological malignancy [1]. Prognosis, progression‐free survival (PFS) and overall survival (OS) improved over the last decades, but myeloma treatment remains challenging, as physicians need to focus on disease pathophysiology, response, preservation of patients' quality of life (QoL) and prevention of severe adverse events (SAEs) [2]. This remains relevant, because elderly patients with symptomatic MM may be affected with additional comorbidities. These correlate with age and are important determinants for therapy selection and influence PFS and OS [3]. The Revised Myeloma Comorbidity Index (R‐MCI) is a MM‐specific comorbidity score for the evaluation of patients' overall health status, in addition to others [3, 4]. Prognostic R‐MCI factors are impaired lung and kidney function, Karnofsky Performance Status (KPS), frailty according to Fried [5] and age (plus cytogenetics, if available) and are used as weighted factors (http://www.myelomacomorbidityindex.org) [3]. Three prognostic groups are discriminated, namely, ‘fit’ (R‐MCI: 0–3), ‘intermediate‐fit’ (R‐MCI: 4–6) and ‘frail’ patients (R‐MCI: 6–9), with substantially different PFS, OS and SAE occurrence [3].

Due to the response to treatment and MM‐related symptoms dissolving, patients' functional status, R‐MCI and QoL may improve over time. Physical activity (PA) has been suggested to improve this further [6]. In cancer patients, PA has been shown to induce various positive effects, most data being available in breast or colon cancer [7, 8, 9]. Patients with haematological cancer may benefit from exercising similarly, although evidence is still rare [10, 11]. Especially for MM patients during induction and subsequent treatment, evidence from randomized controlled trials (RCT) is limited. This is unfortunate, because these patients may benefit from PA to better endure physical deconditioning, prevent cachexia, muscle wasting and fatigue [12, 13, 14], especially because MM treatment is now continuous, rather than time‐restricted [15, 16]. Albeit exercise in patients undergoing treatment demonstrated feasibility and QoL benefits [17], PA is not routinely performed in MM patients [18]. Moreover, no RCTs are currently available (although various ongoing in clinical trials.gov), assessing PA in newly diagnosed (NDMM) or relapsed/refractory (RRMM) patients, nor those on the impact on response, event‐free survival (EFS) [19] and comorbidity scores in physically active versus inactive patients. Our previous analysis of matched active versus inactive MM patients indicated better response, PFS, OS, R‐MCI, and QoL in active patients [6]. Therefore, in this RCT, we compared patients trained to achieve World Health Organization (WHO)–compliant activity [20] (exercise group) to patients with activity as usual (control group). We analysed demographics, comorbidities (osteolytic lesions, recurrent infections, fatigue and depression), induction tolerance, treatment response, ‘timed‐up‐and‐go‐test’ (TUGT), R‐MCI, QoL via ‘Short Form 12’ (SF‐12 [21]), biomarkers and EFS [19]. Training endurance was tracked via smartwatches and diaries. The trainer time assignment during the RCT intervention was also assessed.

## 2 Methods

REAL‐FITNESS was a prospective, monocentric, 1:1 randomized, controlled trial investigating the effects of WHO‐recommended training on exercise tolerance, symptoms and QoL in NDMM patients. Induction treatment with bortezomib–cyclophosphamide–dexamethasone (VCd) was performed after randomization at baseline to exercise versus control groups. Randomization in REAL‐FITNESS included presence of non‐fracture‐prone myeloma bone lesion, as assessed within the interdisciplinary myeloma tumorboard Freiburg (MM‐TB). Randomization was centralized and performed electronically with the use of a Web‐based response system. Patients were randomly assigned to the exercise or control group using a computer‐generated random list created by a trial assistant who was not involved in the enrolment process.

The study was performed in accordance with the Declaration of Helsinki and International Conference on Harmonization Good Clinical Practice. Funding for the trial was provided by the Faculty of Freiburg (program clinical trials) and Comprehensive Cancer Center Freiburg (#3091332001). The university clinic Freiburg (UKF) served as the trial sponsor. An independent ethics committee approved the study (EV 173/20), and all patients provided written informed consent. The trial was registered at drks.de (DRKS00022250).

**FIGURE 1:**
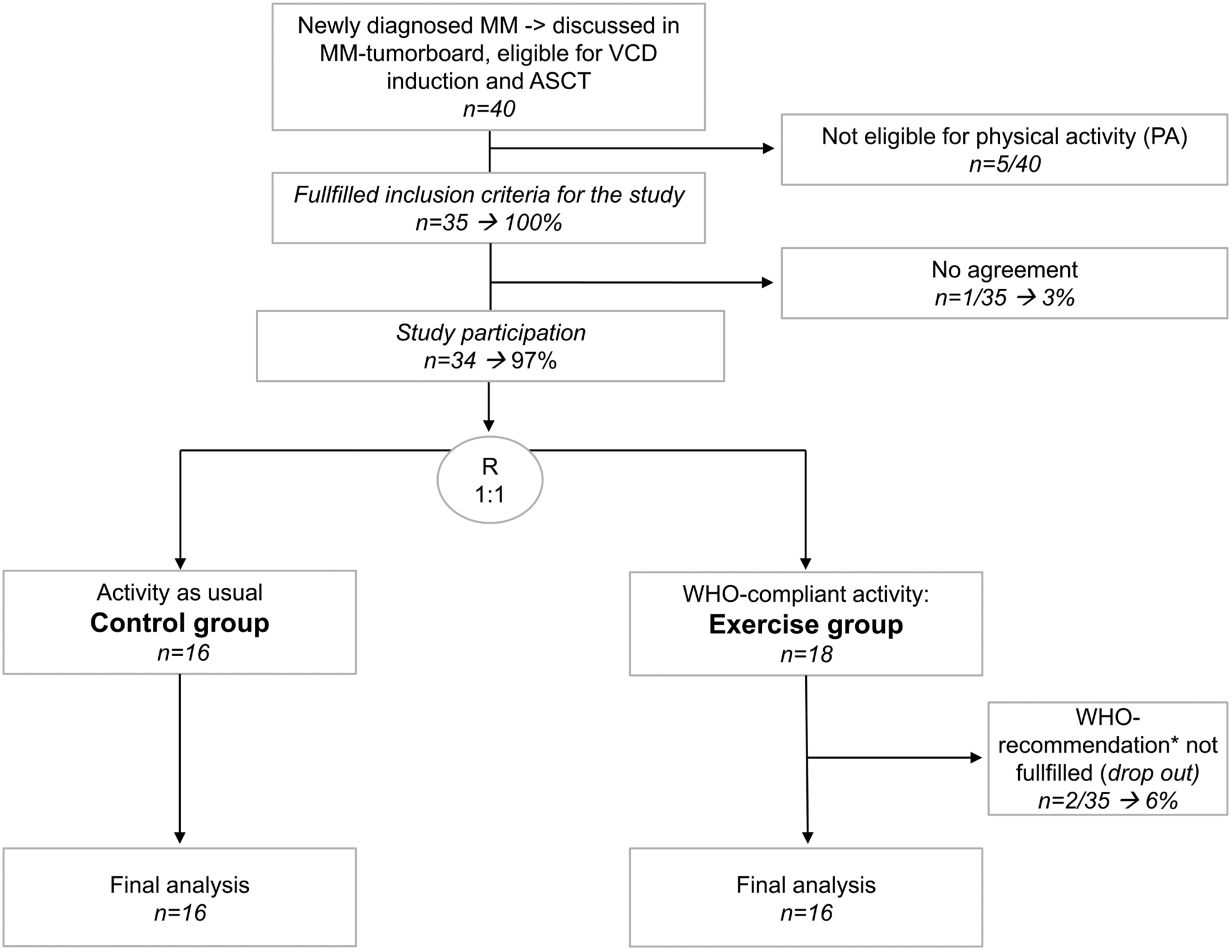
Study design and CONSORT flow diagram of the REAL‐FITNESS‐Study: WHO‐compliant activity (exercise group) versus activity as usual (control group) in newly diagnosed MM patients with VCd induction, patient study inclusion, randomization and group designation. ASCT, autologous stem cell transplantation; control group, activity as usual; exercise group, WHO‐compliant activity; MM, multiple myeloma; n, number; PA, physical activity; R, randomization; VCD, Velcade (V), cyclophosphamide (C), dexamethasone (D); WHO, World Health Organization.

This feasibility study aimed to randomize 40 patients into either an exercise or a control group. The study was performed when COVID started in December 2020, and in the following year, we included 34 patients. A disadvantage of COVID for recruitment was the need to conduct individual 1:1 exercise sessions between a patient and the trainer due to contact restrictions and hygiene regulations. As a result, enrolment was halted after recruiting 40 patients, of whom 34 were included in REAL‐FITNESS (Figure 1 and Table 1).

**TABLE 1:** Patient characteristics at baseline.

Parameter	All patients *n* = 32	Control group *n* = 16	Exercise group *n* = 16
Sex: male/female, *n* (%)	22 (69)/10 (31)	11 (69)/5 (31)	11 (69)/5 (31)
Age (years median; range)	67 (43–89)	62 (43–89)	69 (53–77)
Age (years, mean ± SD)	64.2 (9.8)	61.1 (11.0)	67.3 (7.5)
R‐ISS, *n* (%)			
I/II	8 (25.0)/14 (43.8)	4 (25.0)/7 (43.8)	4 (25.0)/7 (43.8)
III	10 (31.2)	5 (31.2)	5 (31.2)
Type MM, *n* (%)			
IgG	13 (40.7)	7 (43.8)	6 (37.6)
IgA	9 (28.1)	4 (25.0)	5 (31.2)
κ‐/λ‐light chains only	9 (28.1)	4 (25.0)	5 (31.2)
Asecretory	1 (3.1)	1 (6.2)	0 (0.0)
κ‐/λ‐light chains/asecretory	21 (65.7)/ 10 (31.2)/1 (3.1)	10 (62.6)/5 (31.2)/1 (6.2)	11 (68.5)/5 (31.2)/0 (0.0)
Cytogenetics^a^, *n* (%)			
Favourable	19 (59.4)	9 (56.3)	10 (62.5)
Unfavourable	11 (34.4)	5 (31.2)	6 (37.5)
Missing	2 (6.2)	2 (12.5)	0 (0.0)
Marital status, *n* (%)			
Married	24 (75.0)	12 (75.0)	12 (75.0)
Single	8 (25.0)	4 (25.0)	4 (25.0)
Income^b^, *n* (%)			
Medium–high	26 (81.3)	13 (81.3)	13 (81.3)
Low	6 (18.7)	3 (18.7)	3 (18.7)

*Note:* There were no demographic nor treatment differences between control and exercise groups; even gender, marital status and income were well balanced.

Abbreviations: Exercise group with WHO‐compliant activity; control group with activity as usual; Ig = immunoglobulin; MM = multiple myeloma; *n* = number; R‐ISS = Revised International Staging System; κ = kappa; λ = lambda.

^a^ Cytogenetics: favourable = hyperdiploid, t(11;14), normal karyotype, del(13q14); unfavourable = del(17p), t(4;14), t(14;16), t(14;20), hypodiploid, c‐Myc, chromosome 1 aberrations.

^b^ Median‐high income: > 1.025$/month; low income: < 1.025$/month [22].

### 2.1 Primary and Secondary Endpoints

In this monocentric RCT with two parallel arms (exercise vs. control group), evaluating safety and feasibility of exercise in NDMM patients undergoing induction, was the primary objective. To evaluate safety of PA under VCd treatment, all SAEs were documented, and exercise‐related SAEs were recorded separately. The secondary objective was the assessment of exercising efficacy based on the endpoints EFS and R‐MCI, as well as changes in MM‐specific comorbidities, functional tests, biomarkers and QoL. Feasibility of the exercise/WHO training programme was assessed based on patients' willingness to participate, compliance of included exercise patients to fulfil 12 weeks of training during their induction treatment and safety (additional SAEs, bone fractures or other skeletal‐related events [SRE] related to the exercise programme). EFS was defined as the time from randomization to progressive disease (PD), dose reduction, haematological adverse effects (AEs) according to CTCAE grade 4 or non‐haematological AEs according to CTCAE grade ≥3 as described [19]. As feasibility and safety of exercise in NDMM patients was the primary endpoint, no formal sample size calculation was carried out. Instead, we had assessed at the number of patients recruited in other feasibility exercise intervention studies for patients with MM, for example, Larsen et al. (*n* = 30 [17]) and Cenik et al. (*n* = 40 [23]).

We compared trainer assignment time in exercise versus control group (Table 2), patient demographics at baseline (Table 1), comorbidities (osteolytic lesions, fatigue, depression, recurrent infections and anaemia), QoL and related aspects (SF‐12‐questionaire [21]), AEs (treatment tolerance, hospitalization and response to therapy), fitness parameters (TUGT, grip strength (using SAEHAN hydraulic hand dynamometer), R‐MCI‐changes (Table 3) and EFS [19]. Moreover, depression was evaluated by using the Hamilton Depression Scale (HDS using the HDRS17 version of the score, which consists of 17 items, as recommended in guidelines for unipolar depression). Anaemia and fatigue were classified via CTCAE of NCI grade ≥2 and recurrent infections according to CTCAE grade ≥3. Patients were considered to have fatigue, if they reported suffering from general weakness that could not be relieved by rest. Therapy tolerance was defined by nausea, vomiting and/or diarrhoea according to CTCAE criteria ≥1. A biomarker analysis, previously suggested to be of value in trained versus untrained cancer patients [2, 24], was performed, including albumin, creatine kinase (CK), CRP, high‐density lipoprotein (HDL), low‐density lipoprotein (LDL) and pro‐brain natriuretic peptide (proBNP). The recording of parameters at defined points in time is shown in Data S1.

**TABLE 2:** Trainer assignment for RCT in control, exercise and entire patient cohort over 3 VCD induction cycles.

Control group						
	Screening	VCd cycles #1–3			EOT	Σ
		Preparation	Control assessment	Documentation		
Workload/patient	3 h	5.5 h	5.5 h	7 h	3 h	24 h
Σ (*n* = 16 patients)						384 h

Exercise group						
	Screening	VCd cycles #1–3			EOT	Σ
		Preparation	Exercising	Documentation		
Workload/patient	3 h	19 h	19 h	7 h	3 h	51 h
Σ (*n* = 16 patients)						**816 h**

Entire RCT cohort		
32 patients		1200 h

Abbreviations: #1–3, VCd cycles 1–3; ∑, sum; EOT, end of treatment; exercise group with WHO‐compliant activity, control group with activity as usual; *n*, number; RCT, randomized controlled trial; VCd, Velcade (V), cyclophosphamide (C), dexamethasone (D).

**TABLE 3:** ‘REAL‐Fitness’ study parameters at baseline versus at end of treatment (EOT).

	Parameters	Baseline		End of treatment		Treatment effect at EOT (95% CI)	*p* (EOT comparison between both groups)
		Control (*n* = 16)	Exercise (*n* = 16)	Control (*n* = 16)	Exercise (*n* = 16)		
Comorbidities	> 2 Osteolytic lesions, *n* (%)	14 (87.4)	13 (81.3)	14 (87.4)	13 (81.3)	*−0.063 (−0.31 to 0.19)*	*1.00*
	Anaemia (Hb < 10 g/dL), *n* (%)	7 (43.8)	5 (31.3)	10 (62.5)	5 (31.3)	*−0.31 (−0.64 to 0.016)*	*0.16*
	Fatigue (CTCAE grade ≥2), *n* (%)	13 (81.3)	6 (37.5)	12 (75.0)	1 (6.3)	*−0.69 (−0.93 to −0.44)*	*0.0002*
	Comorbidities Recurrent infections, *n* (%)	3 (18.7)	1 (6.3)	1 (6.3)	0 (0.0)	*−0.063 (−0.18 to −0.06)*	*1.00*
	*n* (%)	7 (43.8)	6 (37.5)	7 (43.8)	1 (6.3)	*−0.38 (−0.65 to −0.11)*	*0.037*
	HDRS17 (mean ± SD)	6.0 ± 5.8	5.2 ± 4.8	5.3 ± 5.2	1.2 ± 1.5	*−4.1 (−6.8 to −1.3)*	*0.0055*
Fitness parameter	Weight (mean ± SD; kg)	80.2 ± 12.7	75.8 ± 11.6	80.4 ± 15.0	76.1 ± 14.1	*−4.3 (−14.8 to 6.3)*	*0.42*
	BMI (mean ± SD; kg/m^2^)	26.8 ± 4.3	25.9 ± 3.8	27.0 ± 4.9	25.9 ± 4.1	*−1.1 (−4.4 to 2.1)*	*0.48*
	TUGT (mean ± SD; s)	10.9 ± 4.6	10.6 ± 5.1	11.0 ± 4.8	7.0 ± 2.2	*−4.0 (−6.7 to −1.3)*	*0.0050*
	Grip strength (mean ± SD; lb)						
	Right	77.1 ± 30.6	69.9 ± 20.0	77.3 ± 29.9	79.9 ± 24.6	*2.6 (−17.2 to 22.3)*	*0.79*
	Left	72.6 ± 29.8	63.9 ± 24.4	70.8 ± 30.5	68.9 ± 26.5	*−1.9 (−22.5 to 18.8)*	*0.85*
	R‐MCI (mean ± SD)	4.3 ± 1.6	3.9 ± 2.1	3.4 ± 2.0	2.9 ± 1.9	*−0.6 (−2.0 to 0.8)*	*0.42*
	Fit (0–3), *n* (%)	5 (31.2)	7 (43.8)	9 (56.3)	11 (68.8)		
	Intermediate‐fit (4–6), *n* (%)	10 (62.5)	7 (43.8)	6 (37.4)	4 (24.9)		
	Frail (7–9), *n* (%)	1 (6.3)	2 (12.4)	1 (6.3)	1 (6.3)		
QoL	SF‐12						
	MCS (mean ± SD)	47.4 ± 11.8	45.8 ± 13.0	46.1 ± 11.9	54.6 ± 6.1	*8.4 (1.6 to 15.3)*	*0.017*
	PCS (mean ± SD)	31.8 ± 12.7	40.3 ± 12.3	31.4 ± 10.9	45.4 ± 11.6	*14.0 (5.8 to 22.1)*	*0.0014*
Biomarkers	Albumin (g/dL) (mean ± SD)	3.7 ± 0.7	3.8 ± 0.6	4.0 ± 0.7	4.0 ± 0.6	*0.06 (−0.4 to 0.5)*	*0.81*
	HDL (mg/dL) (mean ± SD)	50.6 ± 21.0	43.4 ± 14.5	*47.2* ± 13.6	51.8 ± 16.8	*4.6 (−6.4 to 15.6)*	*0.40*
	LDL (mg/dL) (mean ± SD)	107.7 ± 41.7	113.3 ± 41.6	119.7 ± 73.4	150.5 ± 39.5	*30.8 (−11.8 to 73.4)*	*0.15*
	CK (U/L) (mean ± SD)	70.3 ± 43.0	59.6 ± 33.0	43.6 ± 22.2	67.7 ± 43.5	*24.1 (−0.8 to 49.1)*	*0.057*
	CRP (mg/L) (median, range) (mean ± SD)	3.3 (1.5; 104.1) 16.7 ± 28.7	4.3 (1.5; 114.1) 13.4 ± 28.1	2.5 (1.5; 19.1) 5.1 ± 5.0	1.5 (1.5; 24.5) *3.6* ± 5.8	*−1.5 (−5.4 to 2.4)*	*0.15*
	proBNP (pg/mL) (median, range) (mean ± SD)	324.5 (44; 4733) 1163.7 ± 1611.8	181.0 (50; 8363) 702.1 ± 2046.8	177.5 (25; 3736) 737.8 ± 1084.7	140.0 (25; 527) 180 ± 141.0	*−557.8 (−116.3 to 0.7)*	*0.53*

*Note:* Treatment effect: difference of means or risk difference (95% CI); exercise minus control. EOT group comparisons via Fisher's exact test; two‐sample *t*‐test; Wilcoxon's two‐sample test (CRP, proBNP).

Abbreviations: BMI, body mass index; CK, creatine kinase; control group, performing activity as usual; CRP, C‐reactive Protein; EOT, end of treatment; exercise group, performing WHO‐compliant activity; Hb, haemoglobin; HDL, high‐density lipoprotein; HDRS17, Hamilton Depression Rating Scale containing 17 items; LDL, low‐density lipoprotein; MCS, Mental Component Scale; complete remission; n, number; PCS, Physical Component Scale; PR, partial remission; proBNP, brain natriuretic peptide; QoL, quality of life; R‐MCI, Revised Myeloma Comorbidity Index; SD, standard deviation; SF‐12, Short Form 12; stable/PD, stable disease or progressive disease; TUGT, timed‐up‐and‐go test; U/L, unit per litre; vgPR, very good partial remission.

### 2.2 Trial Procedures

The inclusion criteria for this RCT required patients to have NDMM and to be reviewed within our MM‐TB (Figure 1). Patients were judged eligible for PA, assessed via physicians of different disciplines as described [25, 26]. Patients with extensive, unstable bone lesions, with a tendency to fracture or inability to walk, were excluded from the study. To ensure comparability, all patients had to receive VCd induction. Between 12/2020 and 11/2021, consecutive NDMM patients who received three cycles of VCd (bortezomib 1.3 mg/m^2^ day (d)1, 8, 15 subcutaneously [sc]; cyclophosphamide 900 mg/m^2^ d1 intravenously [iv]; dexamethasone 40 mg d1, 8, 15 orally [po]; > 70 years: 20 mg; repeated every 21d) were included. Forty patients with NDMM were discussed via our MM‐TB: 35 patients were eligible for PA, of whom 34 consented to participate and 32 were included in the analyses, showing a high willingness to participate (Figure 1).

VCd induction was performed according to the German (DSMM/GMMG) and European Myeloma Network (EMN) study group regimen and guideline‐compliant MM pathways. Cytogenetics were available in 94% of patients. Remission and relapse were defined according to the International Myeloma Working Group criteria [27]. Extend of comorbidities and SAEs were defined via Common Terminology Criteria for adverse effects (CTCAE) of US National Cancer Institute (NCI). The R‐MCI was determined as described [3, 6].

**FIGURE 2:**
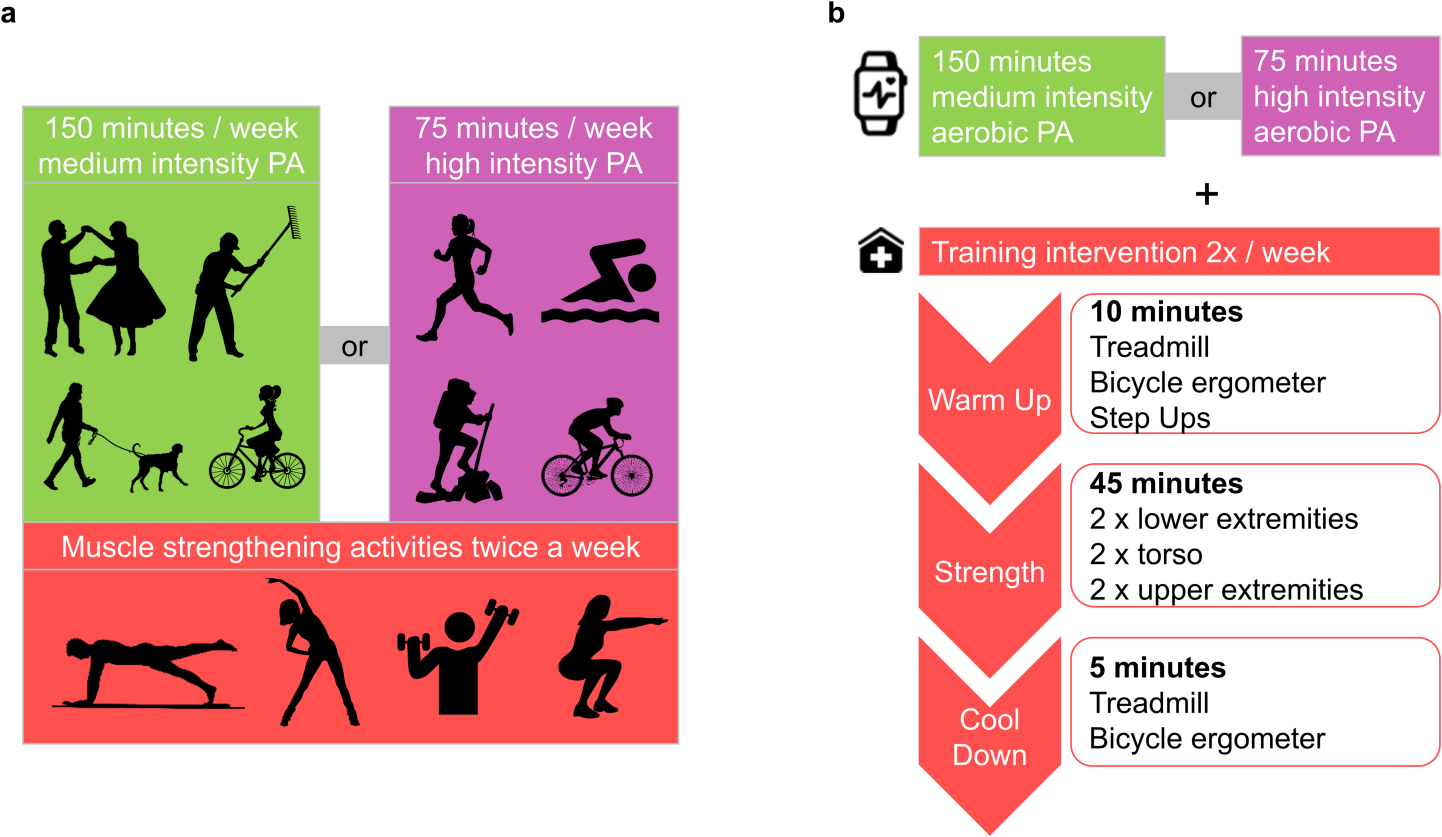
Physical activity. (a) Training was performed in the exercise cohort according to WHO‐recommended guidelines with 150 min of moderate intensity training per week, performed under individual guidance of a certified, MM‐experienced trainer (MDM). The WHO‐based trainings also involved muscle strengthening exercises. (b) Typical training performance for MM patients, involving a warm up‐, strengthening‐ and cool down‐part. WHO, World Health Organization.

Exercise patients received personal trainer–guided exercise sessions according to WHO recommendations: 150 min of moderate aerobic exercising weekly and resistance training of large muscle groups twice a week (Figure 2a) [20]. Aerobic PA was carried out at home on the patients' own responsibility checked via smartwatches (Xiaomi Amazfit Bip) and diaries. All training sessions of at least 10 min were allowed to be recorded in the training diary and were confirmed by reading out the smartwatch being worn. Aerobic training was only considered valid, if the training diary entries corresponded to a heart rate of at least 100, as recorded by the smartwatch provided to all study participants.

Due to the complexity of patients' individual comorbidities and COVID‐19 regulations, strengthening exercises were carried out as supervised individual training sessions (qualified trainer: MDM throughout RCT) on therapy days (d)1, d8 and d15 of each VCd cycle. Training was conducted on an additional day each week to meet WHO recommendations. Trainings were adjusted and progressively intensified based on patients' general condition and MM‐specific symptoms. An exemplary training session is depicted in Figure 2b.

In the exercise group, patients were deemed compliant, if they regularly performed aerobic training at home for at least 150 min per week and took part in the strengthening exercise sessions. In the control group, patients continued their usual level of activity without the requirement to meet WHO training recommendations. The PA of patients randomized to the control group was also assessed using smartwatches and diaries. Due to the extended COVID‐19 pandemic and severe contact restrictions, the legal regulations and hygiene protocols of the UKF needed to be adhered to throughout the entire RCT [28]. The control group was assessed with the same frequency as the exercise group through personal contacts, phone calls and virtual meetings to ensure equal interactive guidance and support for both groups.

### 2.3 Statistical Analysis

Data were analysed using SAS statistical software version 9.4 (SAS Institute Inc., Cary, NC, USA). As feasibility was the primary objective of the trial, no formal sample size calculation was performed. We therefore considered the reported *p* values as descriptive information.

For continuous variables, end‐of‐treatment (EOT) group comparisons were conducted with *t*‐tests for unpaired data, and treatment effects were presented as differences of group means (exercise minus control) with two‐sided 95% confidence limits. In the case of large deviations from the normal distribution, non‐parametric methods were applied, that is, median (range) in addition to mean (SD), and Wilcoxon's two‐sample test.

EOT group comparisons for binary data were performed with Fisher's exact test. As odds ratios cannot always be calculated due to zero entries, treatment effect estimates were presented as risk differences (exercise minus control) with two‐sided 95% confidence limits.

EFS was calculated as time from diagnosis to one of the defined events (progression, death and SAEs) as described [19]. EFS was estimated and displayed using the Kaplan–Meier method and compared with the log‐rank test.

## 3 Results

A total of 40 patients with NDMM were presented in the MM‐TB and were determined to receive VCd induction. Five of them were excluded due to extensive, unstable bone lesions, particularly prone to fractures (Figure 1). The remaining 35 patients were offered to participate in this RCT, of which 34 patients gave their written informed consent (study participation: 97%). The randomization did not lead to entirely balanced groups at baseline. Whereas 16 patients were randomized into the control cohort, 18 patients were in the exercise group, leading to imbalanced group sizes at baseline (Figure 1). In the training group, WHO recommendations were to be met through regular exercise interventions, both in clinics and at home (Figure 2a,b). Two patients did not fulfil WHO recommendations throughout the study period (adherence in intervention group: 16/18; 89%; Figure 1). These two showed exercise activities rates below WHO recommendations via smartwatch‐readouts. Their MM‐ and patient characteristics were no different than the others in the training or control group. Study adherence of the entire cohort was 94% (32/34). After confirmation of significantly lower PA in the control group (below WHO recommendations measured by smartwatches and diaries; Data S2), a total of 32 patients were included into the final analysis (Figure 1). Patients' characteristics at baseline are described in Table 1. The median age of the entire cohort was 67 years. Gender distribution, R‐ISS and cytogenetics were well comparable in both groups. The MM paraprotein type and disease characteristics were likewise similar. We assessed income as medium or high versus low as described [22] and marital status, which proved similar in both groups (Table 1).

With 2×/week trainings over 3 months of induction in 32 patients, this led to 768 encounters between patients and trainer and with 13 repeatedly performed tests to 8553 test repeats. The time spent by the trainer during the RCT amounted to 1200 h. The exact breakdown of the training assignment of the entire cohort is summarized in Table 2. During the entire RCT, the trainer remained the same (MDM). Via smartwatch and diaries, the exercise group proved to be twice as active as the control group (Data S2). With a median of 162‐min aerobic exercising per week and resistance training twice a week in the exercise cohort, our sport programme was feasible for MM patients.

### 3.1 Primary and Secondary Endpoints

Group comparisons of baseline regarding comorbidities, fitness parameters, QoL, AEs, response and biomarkers are summarized in Table 3. The data were well comparable: That is, multiple osteolyses at baseline were apparent in 87.4% versus 81.3%, respectively, and remained similar at EOT.

There were no significant differences in the incidence of recurrent infections and anaemia, neither at baseline nor EOT. Fatigue was more prevalent in control patients at baseline compared to exercise patients. At EOT, significant difference in fatigue remained, which was 12.5 times as high in the control versus exercise group with 75% versus 6%, respectively.

In both cohorts, the mean HDS score, TUGT and R‐MCI were similar at baseline. At EOT, the mean HDS score showed a substantial treatment effect (exercise − control group) of −4.1, providing a statistically significant decrease of depression in the exercise group (*p* = 0.0055). Additionally, the number of patients with depression (HDS > 8 points) at EOT remained the same in the control group with seven patients (43.8%), but significantly declined in the exercise group from six patients at baseline (73.5%) to 1 (6.3%) at EOT.

Although the mean TUGT time did not change within the control group from baseline to EOT, the exercise group lowered their TUGT time by 3.6 s, 34% less than at baseline, leading to a treatment effect of −4.0 between the groups at EOT (*p* = 0.0050).

**FIGURE 3:**
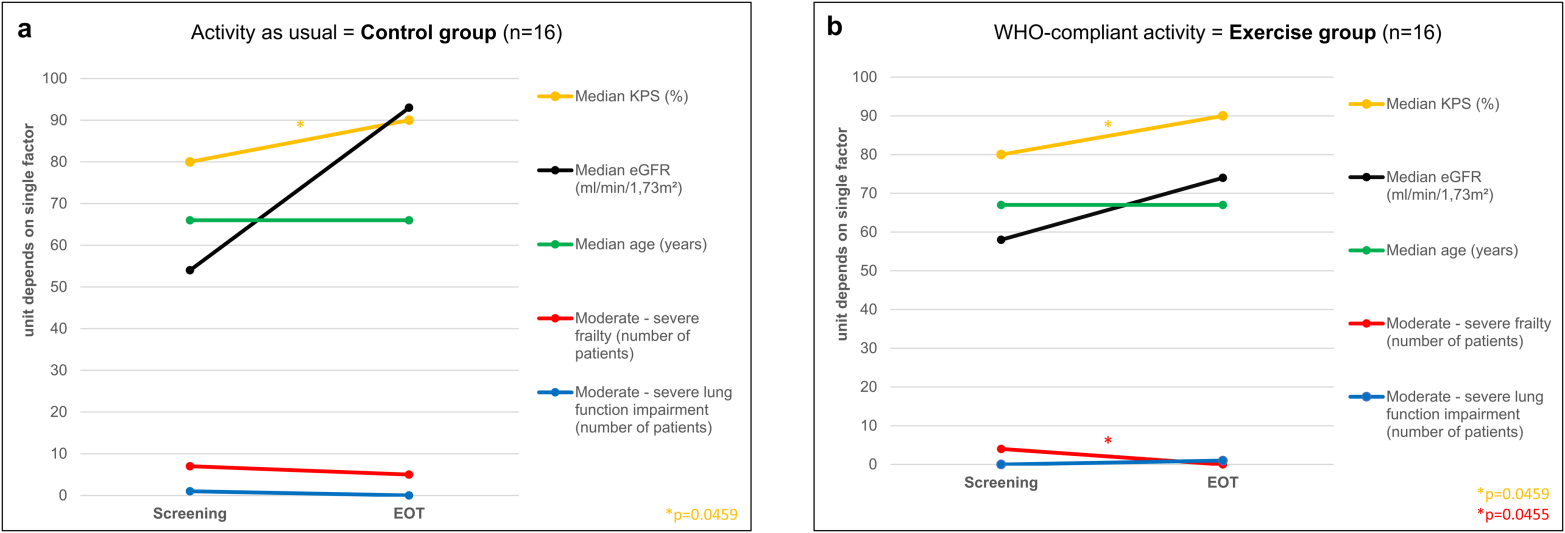
Revised myeloma comorbidity Index (R‐MCI) domains of KPS, eGFR, age, frailty and lung impairment at screening and end of treatment in (a) WHO‐trained = exercise group versus (b) control group with activity as usual. (a) Single factors (KPS, age, frailty, impairment of renal and lung function) are shown for WHO‐trained exercise patients at initial diagnosis and end of treatment (EOT). (b) Single factors [KPS, age, frailty, impairment of renal and lung function] are shown for control patients at initial diagnosis and end of treatment (EOT). eGFR, estimated glomerular filtration rate; EOT, end of treatment; KPS, Karnofsky Performance Status.

The mean R‐MCI decreased by about 1 point from baseline to EOT in both groups. The number of fit, intermediate‐fit and frail patients was also assessed separately in both groups at baseline versus EOT. In exercise patients, the number of fit patients increased from 7 to 11, intermediate‐fit patients declined from 7 to 4, and frail patients declined from 2 to 1 patient, respectively (Table 3). In controls, the number of fit patients increased from 5 to 9, intermediate‐fit patients declined from 10 to 6, and the only frail patient remained frail at EOT (Table 3). Single R‐MCI risk parameters in both cohorts at baseline vs. EOT are displayed in Figure 3: The KPS improved from 80% to 90% in both groups. The eGFR also improved in both under VCd and response to treatment. Likewise, the number of patients who showed moderate to severe frailty decreased in both groups: Whereas the exercise cohort showed significant improvement from 4 to 0 patients, the control group showed less change*,* with frailty in seven patients decreasing to only five patients.

Grip strength at baseline showed no relevant difference. At EOT, grip strength improved in both cohorts, but substantially more in exercise patients (right hand: 73– > 82 lb; left hand: 68– > 72 lb), although a slight improvement was reached in the control group (right hand: 73– > 79 lb; left hand: 68– > 70 lb; Table 3). The mean body‐mass‐index (BMI) at baseline of control versus exercise patients was 26.8 kg/m^2^ versus 25.9 kg/m^2^, respectively. At EOT, the BMI of both groups remained similar (27.0 versus 25.9 kg/m^2^, respectively).

Whereas the Mental Component Scale (MCS) of the SF‐12 questionnaire was similar between the groups at baseline, the exercise group had better Physical Component Scale (PCS) scores at baseline (mean difference of 8.5). At EOT, the MCS and PCS of the control group remained similar to the scores at baseline, whereas the exercise group substantially improved both MCS and PCS (Table 3).

The assessed biomarkers were almost identical in both groups at baseline but showed differences at EOT in both exercise and control patients: albumin, HDL and LDL increased, whereas CRP and proBNP declined. In exercise patients, significant increases in HDL and LDL were observed from baseline to EOT; but no statistically significant differences were found between exercise and control groups at EOT (Table 3).

**FIGURE 4:**
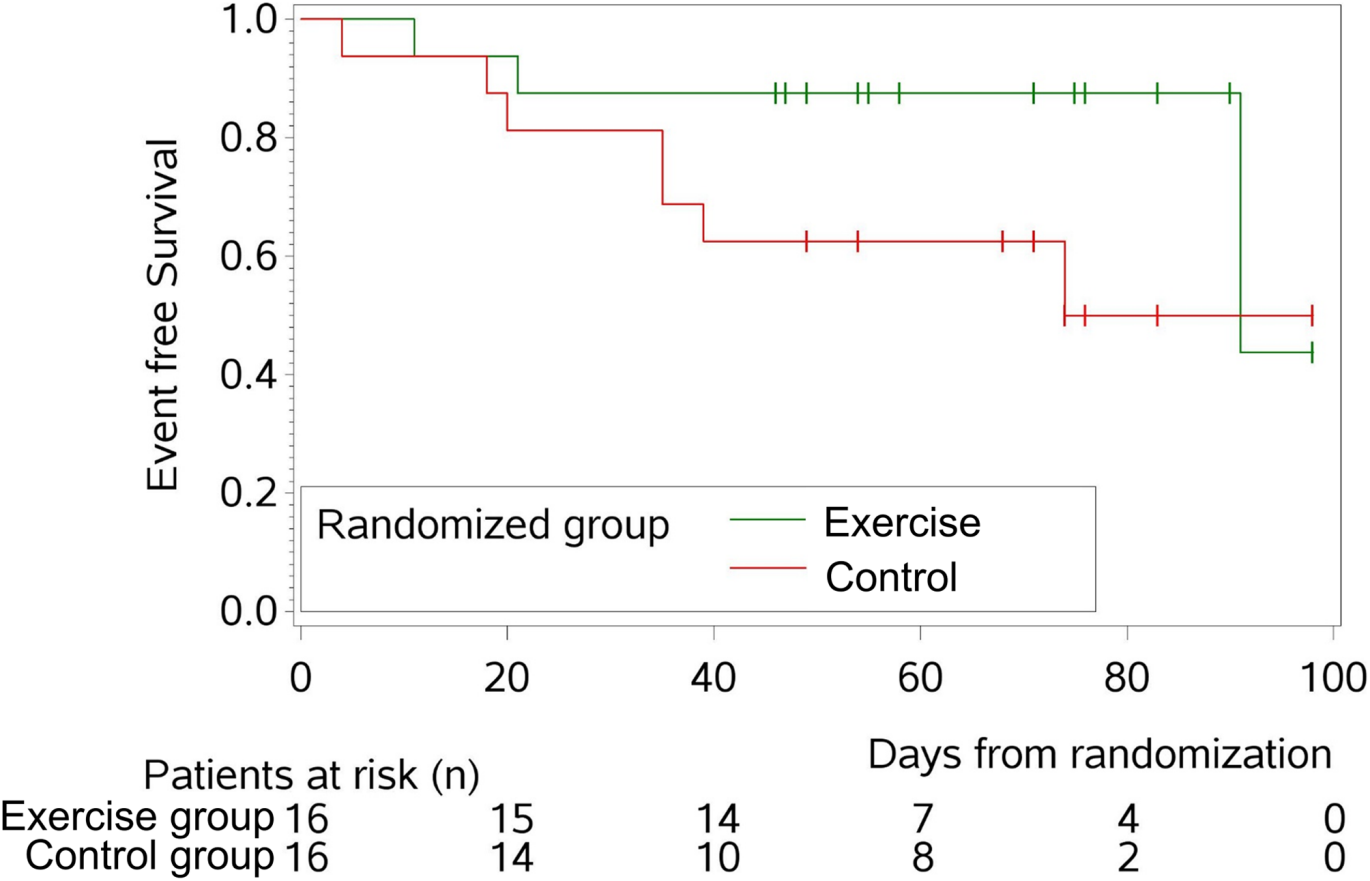
Effects of physical activity on event‐free survival (EFS) for patients receiving VCd induction treatment within cohort of WHO‐trained = exercise group vs. control group. *n*, number; exercise group with WHO‐compliant activity, control group with activity as usual.

Figure 4 displays the EFS for exercise versus control patients with 3 versus 7 events, respectively (more than twice as many events during the 12‐week RCT for control patients, but without statistical significance, *p* = 0.13). The EFS rate after 90 days in exercise versus control patients was 88% (95% CI: 59–97%) and 50% (95% CI: 20–74%), respectively.

### 3.2 Safety

The appearance of SAEs, VCd intolerance, dose adjustments, hospitalization and treatment response were assessed at EOT. SAEs were less frequent in the exercise group (1 vs. 4), likewise therapy intolerance (1 vs. 4) and dose adjustments (1 vs. 6 patients, respectively; Table 4), but without reaching statistical significance. Additionally, the hospitalization rate was lower in the exercise compared to the control cohort (5 vs. 8 patients, respectively). Although there was no significant difference in the duration of in‐patient hospitalization stays, the mean duration of hospitalization was approximately 2 days shorter with 0 ± 7 days for exercise versus 2 ± 15 days for control patients (Table 4). As VCd is almost exclusively an outpatient regimen, hospitalization days were rare. Therefore, differences in hospitalization rates between the two groups were challenging to prove, consistent with a prominently published RCT [29].

**TABLE 4:** Safety of ‘REAL‐Fitness’ study parameters at end of treatment (EOT).

Parameters	Control group (*n* = 16)	Exercise group (*n* = 16)	*p*
AEs, *n* (%)	4 (25.0)	1 (6.3)	*0.33*
Therapy intolerance, *n* (%)	4 (25.0)	1 (6.3)	*0.33*
Dose adjustment, *n* (%)	6 (37.5)	1 (6.3)	*0.083*
Hospitalization, *n* (%)	8 (50.0)	5 (31.3)	*0.47*
Duration (median days ± SD)	2 ± 15	0 ± 7	*0.24*
Treatment response, *n* (%)			
Responder (CR, vgPR, PR)	9	14	*0.65*
Non‐responder (stable/PD)	7	2	*0.25*

Abbreviations: AE, adverse event; CR, complete remission; n, number; PR, partial remission; SD, standard deviation; stable/PD, stable disease or progressive disease; vgPR, very good partial remission.

There were no exercise‐related SAEs or SREs during our training intervention. Therefore, our exercise was safe, especially when it was carried out with the support of a personal trainer.

Our RCT suggested that PA in MM patients may induce positive effects on various MM‐related comorbidities, like fatigue and depression, different fitness parameter (TUGT, grip strength), QoL, cardiovascular and renal biomarkers (albumin, HDL and LDL) and consequently improved the R‐MCI over time. The summary of representative prior studies is displayed in Data S3. In analogy to our RCT, prior interventions combined aerobic and resistance training according to WHO criteria (Figure 2a,b) [17].

## 4 Discussion

Our RCT proved to be a safe and feasible supervised sport intervention during VCd induction in NDMM patients. During the entire treatment period, no sport‐related SAEs or SREs occurred. An important concern regarding PA is the fracture risk with bone lesions, occurring in about 70% in NDMM patients. Nevertheless, prior intervention studies in fracture‐risk cohorts of other osteolytic diseases detected various benefits of sport interventions [15, 30]. In analogy to our RCT, previous interventions combined aerobic and resistance training according to WHO recommendations [17]. Our RCT suggests that PA in MM patients induces beneficial effects on various MM‐related comorbidities like fatigue and depression, different fitness parameters (TUGT and grip strength), QoL (SF‐12 [21]) and cardiovascular and biomarkers (albumin, HDL and LDL) and improves the R‐MCI over time [6, 31, 32]. VCd dose reduction occurred to a lesser extend in exercise patients and EFS improved, suggesting that PA is advantageous for MM patients. No significant differences were observed between the groups in the incidence of recurrent infections or anaemia. Because this feasibility study was not powered to detect differences between exercise and control groups, a *p* value indicating non‐significance does not necessarily mean that there are no differences. Other investigations demonstrated positive effects of exercising on CRP levels, osteolytic lesions and recurrent infections [6, 33].

So far, there are no recommendations regarding type, duration and intensity of PA to induce positive effects on osteolytic lesions. In line, we could not verify bone status improvement, as the 12‐week intervention was too short to detect bone remodelling. Longer intervention periods are necessary to show benefits of PA on osteolytic lesions.

QoL may improve or deteriorate with numbers of received therapy lines in MM patients [34]. PA could improve QoL, despite antimyeloma treatment being performed. Our RCT demonstrated positive effects of PA on patients' QoL (SF‐12), although there were no significant differences in dose reduction, treatment tolerance, hospitalization and SAEs, due to restricted patient numbers within our RCT. As the R‐MCI is influenced by disease and response to treatment, and may reflect therapy‐related changes [26], it was also assessed in exercise versus control patients at baseline and EOT: Although the R‐MCI mean improved from 4 (intermediate fit) to 3 (fit) in exercise patients, this was driven via KPS and ‘frailty status’ improvements. Various investigations have confirmed the positive effect of exercising on patients' frailty [35, 36].

In our RCT, the benefits of PA were also evident through functional fitness parameters: TUGT and grip strength significantly improved in exercise patients, but they declined or remained the same in the control group. These improvements were confirmed in other prospective analyses [23, 37]. Of interest, Cenik et al. postulated that 50% of MM patients participating in regular sport interventions reached the performance status of healthy individuals of their age [23].

Regarding the general health status, both exercise and control groups' BMI remained comparable at baseline and EOT. In line, a current review of Clifford et al. showed no changes of body composition under treatment in 1368 cancer patients. As possible reasons, anabolic resistance, the malignant disease itself and the received therapy were named [38]. For MM patients, maintaining a stable BMI during treatment is desirable, as it indicates good therapy tolerance and performance.

In our previous analysis, we had shown better treatment response in active compared to inactive patients [6]. These results could not be entirely confirmed here, albeit there were more non‐responders (SD and PD) in the control group. Other prior investigations suggested positive effects of exercising on PFS in cancer patients [6, 39, 40], but there are no data regarding EFS in MM patients. Our RCT showed no significant differences in EFS in exercise versus control patients, albeit the latter showed more than twice as many events (3 vs. 7, respectively). In line, Pophali et al. showed positive effects of a sport intervention on EFS in 3.060 lymphoma patients. Interestingly, these effects were observed in patients who were physically active before and at the time of lymphoma diagnosis and continued PA during lymphoma treatment, whereas increased PA after diagnosis did not affect EFS [41].

This analysis was limited by the number of patients and fairly short intervention period, albeit the total number of 768 training encounters (exercise sessions with the personal trainer) and repeated test batteries (Table 3) of 8553 were substantial. As exercise has no ‘standard dose’ and is dependable on the exercising frequency and patients' motivation, we particularly aimed to reduce variability to achieve meaningful results. Effects of different treatment regimes on PA, as recently reported by Gerland et al., who found differences in subgroups receiving different medications during a PA intervention for patients with breast cancer [42], were ruled out by the study design, only allowing patients receiving VCd induction to participate in our study. Patient motivation as an influencing factor was reduced, as muscle strengthening PA was performed as regular guided training intervention during VCd induction. Aerobic PA, that had to be performed at home, was monitored using a combination of ‘exercising diaries’ and smartwatches. This way, two patients who did not fulfil WHO recommendations through additional training at home were identified and excluded from the final analysis. This may be criticized, as it does not respect the intention‐to‐treat principle. We excluded these patients, because this feasibility study was primarily about generating hypotheses rather than proving efficacy. In order to prevent reduced adherence, which often troubles PA trials, we committed an extensive amount of time per patient (Table 2), achieving a high adherence of 94%.

In conclusion, our results demonstrate the feasibility and safety of a guided exercise intervention programme during induction treatment. This study suggests numerous positive effects of PA but lacks statistical power due to our RCT size. In line with other studies, we recommend integrating PA into routine care and encouraging MM patients to exercise [8]. Our innovation strategies for MM may not only focus on better disease control and longer survival but also on how exercise interventions can be routinely integrated into cancer care. As follow‐up projects, we have assessed the levels of PA before and after the MM diagnosis, whether WHO exercise recommendations are met and how many patients are willing to participate in sport intervention programmes in over 200 patients with MM [43]. In another prospective study, we are investigating MM patients undergoing rehabilitation after autologous stem cell transplantation, in particular whether the assessment tools of this RCT are useful to predict rehabilitation outcome and patients continue to exercise after finishing rehabilitation. Studies in MM patients, when searched via PubMed terms of ‘cachexia AND muscle wasting AND sarcopenia AND exercise’, are yet missing, speaking for this feasibility study. There are planned trials, including in Germany, where sport interventions with longer training periods will be offered to larger MM cohorts. The results of this feasibility trial are certainly informative for these follow‐up trials.

## Author Contributions

Conceptualization: Monika Engelhardt, Mandy‐Deborah Möller, Ralph Wäsch and Gabriele Ihorst. Data curation: Mandy‐Deborah Möller. Formal analysis: Monika Engelhardt, Esther Dreyling, Jan Räder and Mandy‐Deborah Möller. Investigation: Mandy‐Deborah Möller and Monika Engelhardt. Methodology: Mandy‐Deborah Möller, Monika Engelhardt, Jan Räder, Ralph Wäsch and Gabriele Ihorst. Project administration: Monika Engelhardt. Resources: Monika Engelhardt and Ralph Wäsch. Supervision: Monika Engelhardt. Validation: Monika Engelhardt, Gabriele Ihorst and Mandy‐Deborah Möller. Visualization: Monika Engelhardt, Gabriele Ihorst, Esther Dreyling, Jan Räder and Mandy‐Deborah Möller. Writing – original draft: Monika Engelhardt, Esther Dreyling, Jan Räder and Mandy‐Deborah Möller. Writing – review and editing: Monika Engelhardt, Esther Dreyling, Jan Räder, Gabriele Ihorst, Sina Wenger, Antonia Pahl, Jann Arends, Georg Herget, Peter Deibert, Ralph Wäsch. All authors approved the final version to the manuscript.

## Ethics Statement

The study is approved by EV University Clinic Freiburg (UKF) EV 173/20.

## Consent

Patients consented to participate in the study in writing before inclusion in the study. Information contained in the study is kept confidential, all identifiers have been removed prior to submission for publication.

## Conflicts of Interest

The authors declare no conflicts of interest.

## Supporting information


**Data S1** Parameter recording at defined points in time.

**Data S2** Physical activity (minutes per week) in control vs. exercise group (measured via Smartwatch Xiaomi Amazfit Bip).

**Data S3** Review of the literature of sport intervention studies in cancer patients.

## Data Availability

The data that support the findings of this RCT are available from the corresponding author upon reasonable request.
