# Supplementary material for: A Randomized Controlled ‘REAL‐FITNESS’ Trial to Evaluate Physical Activity in Patients With Newly Diagnosed Multiple Myeloma
Source: J Cachexia Sarcopenia Muscle. 2025 Apr 8;16(2):e13793. doi: 10.1002/jcsm.13793 (PMC11976162; doi:10.1002/jcsm.13793)
Supplement: Supplementary file 1 — Data S1 Parameter recording at defined points in time. [file JCSM-16-e13793-s002.docx]

**Supplementary material 1. Parameter recording at defined points in time**

| **Data assessment parameters** | **Screening** | **VCd cycle 1 - 3 (21 days)** | | | **EOT**  **(7 days post)** |
| --- | --- | --- | --- | --- | --- |
|  |  | **Day 1** | **Day 8** | **Day 15** |  |
| VCd induction treatment | X | X | X | X |  |
| Bortezomib s.c. |  | X | X | X |  |
| Cyclophosphamide i.v. |  | X | X | X |  |
| Dexamethasone p.o. |  | X | X | X |  |
| Eligible for PA and informed consent | X |  |  |  |  |
| Randomization | X |  |  |  |  |
| Sport intervention / training |  | X | X | X |  |
| Patient characteristics | | | | | |
| 1. Demographics | X |  |  |  |  |
| 1. Medical history | X |  |  |  |  |
| 1. MM parameters | X |  |  |  |  |
| Health status | | | | | |
| 1. Height, weight, Body mass index | X | X | X | X | X |
| 1. Vital signs | X | X | X | X | X |
| 1. Laboratory parameters | X | X |  |  | X |
| 1. Karnofsky performance status | X |  |  |  | X |
| 1. Physical examination | X |  |  |  | X |
| 1. Comorbidities | X |  |  |  | X |
| 1. Quality of life (SF-12) | X |  |  |  | X |
| 1. Revised Myeloma Comorbidity Index | X |  |  |  | X |
| 1. Biomarkers | X |  |  |  | X |
| 1. Grip strength | X |  |  |  | X |
| 1. Timed up and go test | X |  |  |  | X |
| 1. Adverse events |  | X | X | X | X |
| 1. Response, EFS, OS |  |  |  |  | X |
