# Supplementary material for: A Randomized Controlled ‘REAL‐FITNESS’ Trial to Evaluate Physical Activity in Patients With Newly Diagnosed Multiple Myeloma
Source: J Cachexia Sarcopenia Muscle. 2025 Apr 8;16(2):e13793. doi: 10.1002/jcsm.13793 (PMC11976162; doi:10.1002/jcsm.13793)
Supplement: Supplementary file 2 — Data S2 Physical activity (minutes per week) in control vs. exercise group (measured via Smartwatch Xiaomi Amazfit Bip). [file JCSM-16-e13793-s003.docx]

**Supplementary material 2. Physical activity (minutes per week) in control vs. exercise group (measured via Smartwatch Xiaomi Amazfit Bip®)**

|  | **Control group (n=16 patients)** | |
| --- | --- | --- |
| **Patient** **#** | **Median*** | **Min. - Max.** |
| **1** | 42 | 12 - 63 |
| **2** | 46 | 29 - 78 |
| **3** | 77 | 34 - 111 |
| **4** | 68 | 35 - 97 |
| **5** | 51 | 18 - 82 |
| **6** | 59 | 21 - 64 |
| **7** | 97 | 60 - 129 |
| **8** | 67 | 43 - 83 |
| **9** | 54 | 41 - 67 |
| **10** | 67 | 26 - 87 |
| **11** | 91 | 68 - 125 |
| **12** | 61 | 41 - 80 |
| **13** | 79 | 59 - 92 |
| **14** | 88 | 67 - 99 |
| **15** | 102 | 84 - 131 |
| **16** | 39 | 23 - 48 |
| **Median**** | **61 (12 - 129)** | |

|  | **Exercise group (n=16 patients)** | |
| --- | --- | --- |
| **Patient** **#** | **Median*** | **Min. - Max.** |
| **17** | 174 | 153 - 181 |
| **18** | 183 | 161 - 198 |
| **19** | 157 | 151 - 169 |
| **20** | 190 | 179 - 212 |
| **21** | 166 | 160 - 183 |
| **22** | 157 | 156 - 160 |
| **23** | 177 | 162 - 187 |
| **24** | 172 | 159 - 184 |
| **25** | 153 | 150 - 158 |
| **26** | 181 | 164 – 206 |
| **27** | 182 | 155 - 189 |
| **28** | 169 | 153 - 177 |
| **29** | 176 | 166 - 191 |
| **30** | 184 | 170 - 195 |
| **31** | 165 | 157 - 173 |
| **32** | 158 | 151 - 169 |
| **Median**** | **162 (150 - 212)** | |

*: of minutes per week; **: of median* of all patients within exercise and control groups
