# Supplementary material for: A Randomized Controlled ‘REAL‐FITNESS’ Trial to Evaluate Physical Activity in Patients With Newly Diagnosed Multiple Myeloma
Source: J Cachexia Sarcopenia Muscle. 2025 Apr 8;16(2):e13793. doi: 10.1002/jcsm.13793 (PMC11976162; doi:10.1002/jcsm.13793)
Supplement: Supplementary file 3 — Data S3 Review of the literature of sport intervention studies in cancer patients. [file JCSM-16-e13793-s001.docx]

**Supplementary material. 3. Review of the literature of sport intervention studies in cancer patients**

| Literature | Study design | Cancer | n / study | Endpoint(s) | Results and summary of study results |
| --- | --- | --- | --- | --- | --- |
| Fitness tests & comorbidity scores | | | | | |
| Cenik 2020 | P, MoC | MM | n=40 | Evaluation of PA and fitness tests | 50% reached levels of healthy individuals in TUGT & grip strength |
| Persoon 2017 | RCT, MuC | HM | n=109 | Effects on grip strength | No differences in grip strength |
| Seefried 2020 | P, MoC | MGUS | n=15 | TUG, grip strength | Grip strength + TUGT improved after 3 and 6 months |
| Möller 2020 | R, MoC | MM | n=106 | R-MCI; comorbidities, QoL, PFS, OS | Positive effects of PA on R-MCI, comorbidities, QoL, PFS+OS |
| Summary: Improved fitness tests | | | | | |
| Safety and feasibility | | | | | |
| Christensen 2018 | RCT, MoC | Colon car | n=50 | Under treatment hospitalization | Safety & feasibility confirmed; reduced in-patient stays |
| Coleman 2003 | RCT, MoC | MM | n=24 | PA under treatment (ASCT) | Feasibility & positive effects confirmed |
| Koutoukidis 2020 | RCT, MoC | MM | n=131 | Feasibility of PA; fatigue after therapy | No AEs; PA reduces fatigue |
| Larsen 2019 | RCT, MoC | MM | n=30 | Safety & feasibility, AEs | Safety & feasibility confirmed; no SAEs |
| Lazzari 2021 | Review | Multiple | n=940 / n=14 | Safety & feasibility of PA | Safety & feasibility confirmed; no SAEs |
| Newton 2018 | RCT, MuC | Prostate | n=866 | Feasibility & effects on osteolyses | Safety confirmed in patients with osteolytic lesions |
| Summary: Safety and feasibility confirmed; no SAEs | | | | | |
| Comorbidities and quality of life (QoL) | | | | | |
| Benedetti 2018 | Review | Osteoporosis | n=n.a. / n=25 | Changes in bone status | Positive effects of PA on bone density |
| Bernard 2019 | RCT, MuC | MM | n=48 | Biomarker (CRP, ESR) in responders | CRP predictive factor for therapy response |
| Buffart 2018 | Review | Multiple | n=4519 / n=34 | Fatigue, strength and QoL | Improvement of fatigue, strength and QoL |
| Coleman 2012 | RCT, MoC | MM | n=187 | Effects of PA on fatigue | Minimal effects on fatigue |
| Ferioli 2018 | Review | Multiple | n=n.a. / n=248 | PA on comorbidities and depression | Positive effects on the incidence of AEs |
| Islam 2018 | Review | HM | n=n.a / n=55 | Effects of PA on QoL | Positive effects of PA |
| Jordan 2014 | Cross-sectional, MuC | MM | n=154 | Depression, fatigue, QoL, bone status | Positive effects on depression, fatigue, QoL and bone status |
| Kang 2019 | Cross-sectional, MoC | MM | n=110 | Identification of QoL under treatment | Depression greatest predictive factor |
| Shapiro 2021 | Review | MM | n=9 | Effects of lifestyle on well-being | Positive effects of PA on fatigue, depression and QoL |
| Steffens 2019 | Review | Solid tumors | n=806 / n=17 | Preoperative PA on hospitalization | Preoperative exercising reduces duration of in-patient stay |
| Summary: Positive effects on comorbidity and QoL | | | | | |
| Response, adverse events (AEs) and survival | | | | | |
| Akdeniz 2021 | R, MoC | Breast ca | n=196 | PFS | PA minimizes risk for PD |
| Courneya 2015 | RCT, MuC | Lymphoma | n=122 | Effects of PA on PFS | PA improves PFS |
| Guercio 2019 | Cohort, P | Colon,Leukemia | n=1.218 | Effects of PA on PFS + AEs | Significant correlation between PA and incidence of PD and of AEs |
| Pophali 2018 | R, MoC | Lymphoma | n=3.060 | EFS | PA before diagnosis and under treatment improves EFS |
| Summary: Lack of data | | | | | |

**Abbreviations:** R: retrospective, P: prospective study, MoC: monocentric; MuC: multi-center study; HM: hematological malignancies; ca: cancer; BMI: Body-Mass-Index; CRP: C-reactive protein; EFS: event-free survival;

MM: multiple myeloma; n: number; PA: physical activity; PD: progressive disease; PFS: progression-free survival; RCT:randomized controlled trial; R-MCI: Revised Myeloma Comorbiditiy Index; TUGT: Timed Up and Go test;

ESR: Erytrocyte sedimentation rate
